# Embryonic cholesterol esterification is regulated by a cyclic AMP-dependent pathway in yolk sac membrane-derived endodermal epithelial cells

**DOI:** 10.1371/journal.pone.0187560

**Published:** 2017-11-21

**Authors:** Siou-Huei Wang, Han-Jen Lin, Yuan-Yu Lin, Yu-Jen Chen, Yu-Hui Pan, Cheng-Ting Tung, Harry John Mersmann, Shih-Torng Ding

**Affiliations:** 1 Department of Animal Science and Technology, National Taiwan University, Taipei City, Taiwan; 2 Department of Animal Science and Biotechnology, Tunghai University, Taichung City, Taiwan; 3 Institute of Biotechnology, National Taiwan University, Taipei City, Taiwan; Universite Clermont Auvergne, FRANCE

## Abstract

During avian embryonic development, endodermal epithelial cells (EECs) absorb yolk through the yolk sac membrane. Sterol O-acyltransferase (SOAT) is important for esterification and yolk lipid utilization during development. Because the major enzyme for yolk sac membrane cholesteryl ester synthesis is *SOAT1*, we cloned the avian *SOAT1* promoter and elucidated the cellular functions of *SOAT1*. Treatments with either glucagon, isobutylmethylxanthine (IBMX), an adenylate cyclase activator (forskolin), a cAMP analog (dibutyryl-cAMP), or a low glucose concentration all increased *SOAT1* mRNA accumulation in EECs from Japanese quail, suggesting that *SOAT1* is regulated by nutrients and hormones through a cAMP-dependent pathway. Activity of protein kinase A (PKA) was increased by IBMX, whereas co-treatment with the PKA inhibitor, H89 negated the increase in PKA activity. Cyclic AMP-induced EECs had greater cholesterol esterification than untreated EECs. By promoter deletion and point-mutation, the cAMP-response element (-349 to -341 bp) was identified as critical in mediating transcription of *SOAT1*. In conclusion, expression of *SOAT1* was regulated by a cAMP-dependent pathway and factors that increase PKA will increase *SOAT1* to improve the utilization of lipids in the EECs and potentially modify embryonic growth.

## Introduction

During avian embryonic development, nutrients are mainly absorbed from yolk by the endodermal epithelial cells (EECs) in the yolk sac membrane (YSM). Unlike mammals, development of avian embryos relies exclusively on yolk. For the chicken industry, there are two considerable risks of death during egg incubation. One is when no capillary system develops in eggs at embryonic day (ED) 7 and the second risk happens at ED18 to 20 resulting from inappropriate temperature or humidity, poor ventilation, or because of genetic-related malnutrition.

The most abundant lipoprotein in yolk is very low density lipoprotein (VLDL) representing up to 66% of yolk dry matter. Cholesterol is estimated as 5.2% of total yolk lipids [1]. Approximately 68% of lipids in yolk are absorbed during the late stages of egg incubation. Sterol O-acyltransferase (SOAT), also called acyl-coenzyme A: cholesterol acyltransferase (ACAT), is responsible for the esterification of cholesterol with a long-chain fatty acid, which is important for lipid utilization during embryonic development [2–4]. The increased concentration of esterified cholesterol (cholesteryl ester, CE) in YSM during late stages of embryogenesis has triggered much interest. The concentration of CE increases from 3.3% to 6.9% of total lipids in the chicken YSM during ED 13 to ED21; however, the CE level in yolk remains constant [5, 6]. The importance of SOAT1 in conversion of free cholesterol to less polar CE is to provide better availabilities of cholesterol for VLDL packaging. The lipid would then be rapidly transported and stored in embryonic livers. Hence, the CE levels in embryonic liver increased from 33.9 to 70.2% of total lipids during ED 13 to ED21 [2, 5, 6].

**Fig 1:**
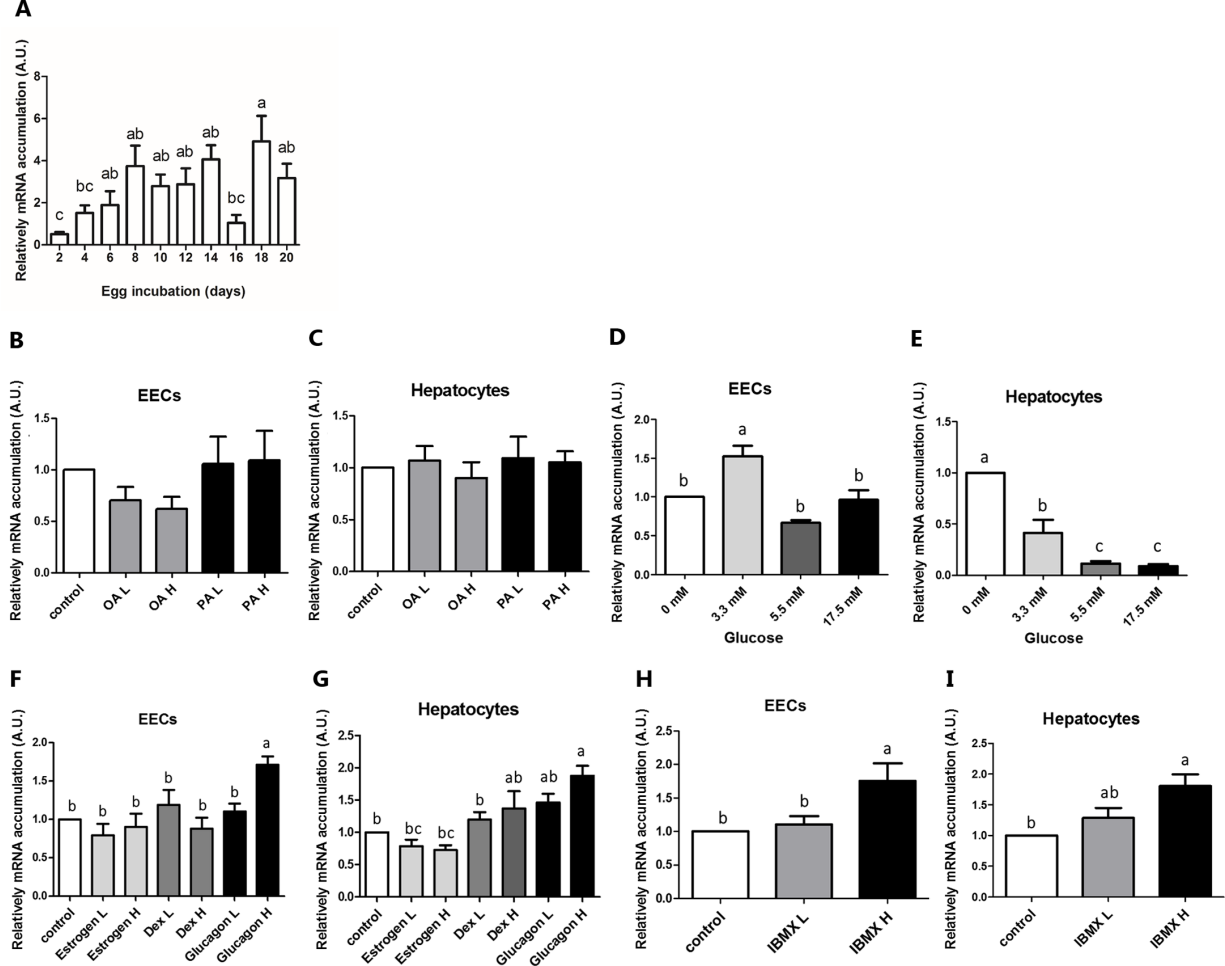
Effects of nutrients and hormones on *SOAT1* mRNA accumulation in EECs and hepatocytes. (A) The mRNA accumulations of *SOAT1* during embryogenesis in YSM of Japanese quail. (B-I) EECs and hepatocytes were treated for 24 hours with different concentrations of a fatty acid, OA (oleic acid) or PA (palmitic acid); L(low) = 10 μM; H (high) = 100 μM, or glucose (3.3, 5.5 or 17.5 mM), or hormones (estrogen L = 1.8 nM, H = 18 nM; Dex L = 1 μm, H = 10 μm, glucagon L = 10 nM, H = 100 nM; IBMX L = 0.05 mM, H = 0.5 mM). *SOAT1* mRNA accumulation was measured by Real-Time PCR and normalized to *β-actin* mRNA accumulation. Data were expressed as mean ± S.E.M. (n = 6) Statistical significance was determined by one-way analysis of variance. Tukey‘s test was used to evaluate differences between means. Control value was set as 1. Different letters indicate a significant difference (P≤0.05).

In mammals or zebrafish, the SOAT family has two subtypes, SOAT1 and SOAT2. Because only SOAT1 is transcribed in YSM during the final week of Japanese quail embryonic development, we suggested that *SOAT1* may have a significant role at late stages of development (Fig 1A). Previous reports suggested that function of this enzyme can be regulated by multiple nutritional factors. The activity of *SOAT1* is increased by dietary cholesterol supplementation in young chick liver [7]. The activity of liver *SOAT1* is inhibited by dietary n-3 polyunsaturated fatty acids in rabbits [8]. The function of mammalian SOAT1 is activated by cholesterol, oxysterols, cholestyramine and various plant sterols [9–11]. Therefore, the functionality of the *SOAT1* enzyme is regulated by nutrients, but the regulatory mechanism is unknown.

To reveal promoter regulation of *SOAT1* transcription, we predicted the possible regulatory binding elements on the *SOAT1* promoter sequence using Genomatix software. Many binding regions were revealed, including cAMP-response elements (CRE, TGAA; ACGT; TGACG), ccaat/enhancer element (TTGAGCAA), estrogen response elements (AACAACGT; CAAGGACA), glucocorticoid related element (GAACA), sterol regulatory element (CTCCCCCCAC) and carbohydrate response element (ACGTG). Therefore, we suggested that treatment with either glucagon, isobutylmethylxanthine (3-isobutyl 1-methylxanthine, IBMX), a nonselective phosphodiesterase inhibitor that increases intracellular cyclic AMP (cAMP) concentration [12], estrogen, dexamethasone, fatty acids (palmitic/ oleic acid) or glucose would target their individual response element to trigger *SOAT1* transcription and influence cholesterol esterification in culture systems.

The abundant mRNA accumulation of *SOAT1* in YSM during later stages of egg incubation indicated the importance of this enzyme in mediating nutrient transfer from yolk to embryos in avian species. The current study was to investigate possible mechanisms by which nutrients and hormones regulate the avian *SOAT1*.

## Materials and methods

All animal studies were approved by the Institutional Animal Care and Use Committee (IACUC) of the National Taiwan University. The IACUC Approval No: NTU-103-EL-113.

### 1. Culture of endodermal epithelial cells and primary hepatocytes from Japanese quail

Isolation of endodermal epithelial cells (EECs) and the culture system were modified from the published procedure [13]. In short, yolk sac membrane tissues from day 5 embryos were treated with collagenase to partially digest the extracellular matrix and facilitate cell isolation [14]. We collected 6 YSM (from 6 embryonic day five embryos) to isolate EECs and pooled as one sample for the experiment. The total number of fertilized eggs and newly-born quails for mRNA detection during development were 54 fertilized eggs and 13 newly-born quails. The sum of embryonic day 5 embryos we used for EECs culture system was 210 fertilized eggs. All the mature quails were purchased from a local farm located at Taoyuan City 327, Taiwan (R.O.C.). EECs were cultured in DMEM/ F12 (12400–024, pH 7.4, Gibco, Waltham, MA) with 10% new born calf serum (16010–159, Gibco) and 1% Pen-Strep Ampho. solution (03-033-1B, Biological Industries, Cromwell, CT). Hepatocytes were isolated from 7 to 10 day-old Japanese quail by procedures modified from previous published protocols [15, 16]. Japanese quail were sacrificed with CO_2_ and liver tissue was gently perfused from the hepatic vein using a scalp-vein set. Perfusion was with the sequential addition of 50 mL of buffer I (154 mM NaCl, 20 mM HEPES, 5.6 mM KCl, 5 mM glucose, 25 mM NaHCO_3_, pH 7.2), 50 mL of buffer II (152.5 mM NaCl, 19.8 mM HEPES, 5.5 mM KCl, 5 mM glucose, 24.8 mM NaHCO_3_, 0.1 mM EGTA, pH 7.2) and 50 mL of buffer III (152.5 mM NaCl, 19.8 mM HEPES, 5.5 mM KCl, 5 mM glucose, 24.8 mM NaHCO_3_, 0.5 μM CaCl_2_·2H_2_O, pH 7.2). This was followed by perfusion with 25 mL of buffer IV (152.5 mM NaCl, 19.8 mM HEPES, 5.5 mM KCl, 5 mM glucose, 24.8 mM NaHCO_3_, 0.5 μM CaCl_2_·2H_2_O, 250 units/ mL collagenase (C6885-1G, Sigma-Aldrich, St. Louis, MO, USA, pH 7.2). The perfused livers were carefully removed and membranes and blood vessels were dissected away. The remaining liver was minced and digested in another 25 mL of perfusion buffer IV at 37°C in a shaking water bath at 175 rpm for 15 minutes. The hepatocytes were filtered through 40 μm mesh and centrifuged at 130 x g for 2 minutes at room temperature. The supernatant fraction was removed then 10 mL of RBC lysis buffer (155 mM NH_4_Cl, 10mM KHCO_3_, 0.1 mM EDTA) was added for 10 minutes followed by 10 mL of DMEM (12800–017, pH 7.4, Gibco) to block the lysis activity. To collect the hepatocytes, the preparation was centrifuged at 130 x g for 2 minutes at room temperature. Pellets were re-suspended and washed twice with 25 mL buffer V (120 mM NaCl, 10 mM HEPES, 0.9 mM CaCl2·2H2O, 6.2 mM KCL, 0.1% w/v albumin, pH 7.2) and centrifugation. Finally, the pellets were suspended in 10 mL DMEM containing 10% FBS (04-001-1A, Biological Industries) and hepatocytes were seeded onto 24-well plates (1.5 x 10^5^ cells/ cm^2^). Quail’s livers were used to isolate hepatocytes for the experiment and one animal was referred as one sample. Twenty quails of 7 to 10 day-old were used in preparing the primary hepatocyte cells for the experiment. Hepatocytes and EECs were treated with different nutrients (glucose or fatty acids), hormones (estrogen (E2758, Sigma-Aldrich, St. Louis, MO, USA), dexamethasone (D4902, Sigma-Aldrich), glucagon (G2044, Sigma-Aldrich)), or IBMX (I5879, Sigma-Aldrich), forskolin (11018, Cayman, Michigan, USA) or dibutyryl cyclic-AMP (db-cAMP, D0627, Sigma-Aldrich), to increase cellular cAMP concentration, and H89 (10010556, Cayman) as a potent PKA inhibitor.

**Fig 2:**
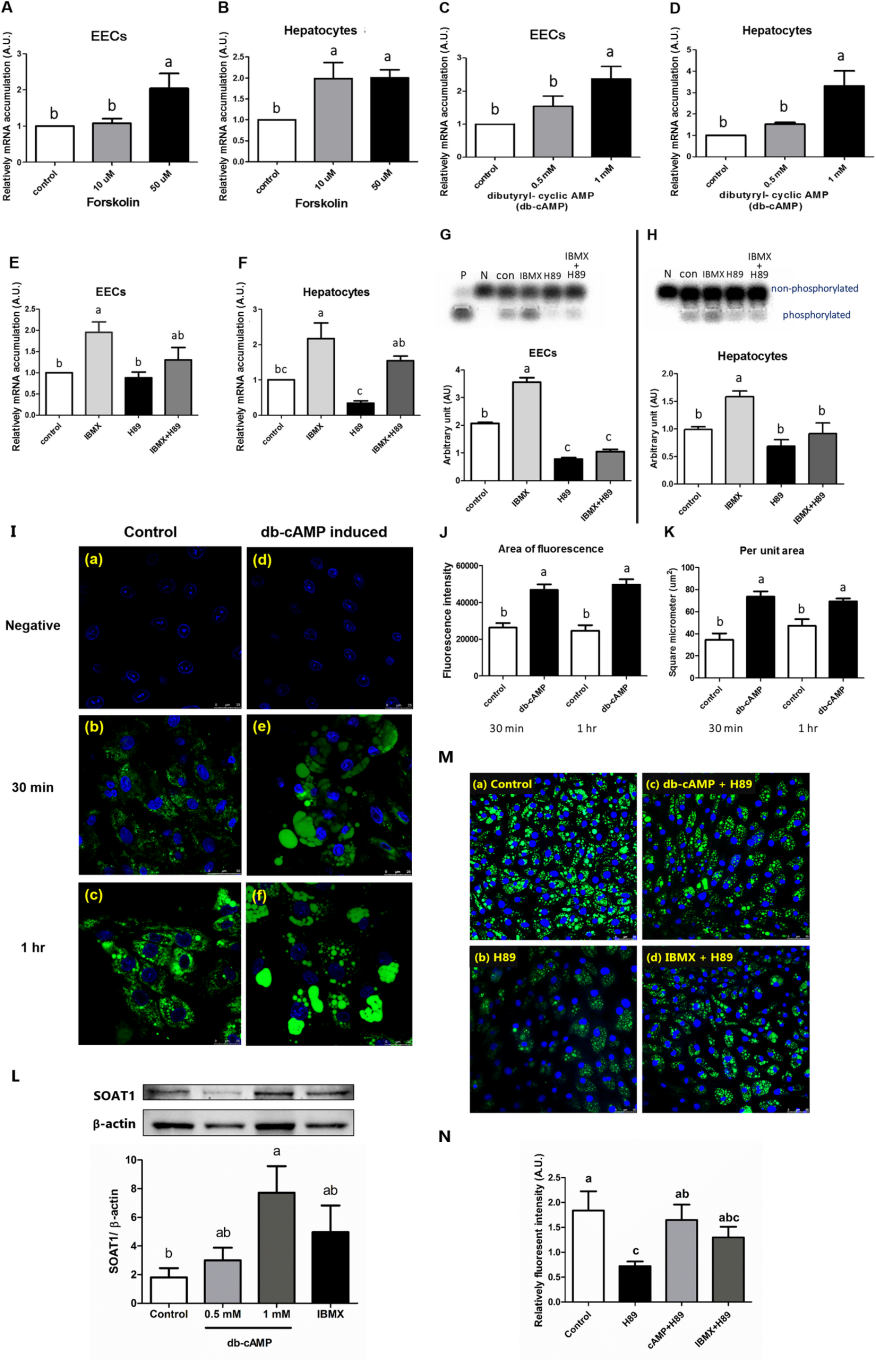
Positive regulation of transcription and enzymatic function of *SOAT1*. (A-D) Effects of forskolin and dibutyryl-cyclic AMP (db-cAMP) on *SOAT1* mRNA accumulations in EECs and hepatocytes. EECs and hepatocytes were treated with different concentrations of forskolin or db-cAMP for 24 hours. *SOAT1* mRNA accumulation was measured and normalized to *β-actin* mRNA. (n = 4) (E-F) H89 inhibited IBMX-induced *SOAT1* mRNA accumulations in EECs and hepatocytes. *SOAT1* mRNA accumulation was increased by 0.5 mM IBMX treatment for 24 hours, whereas the PKA inhibitor, H89 (10 μM) abolished the IBMX-mediated increase of *SOAT1*. (n = 6) (G-H) The IBMX treatment increased PKA activity in EECs and hepatocytes. The cells were treated with 0.5 mM IBMX with or without 10 μM H89 for 24 hours. The cell lysates were assayed for PKA activity using the PepTag assay. Typical gel patterns are represented (three independent experiments) for EECs and hepatocytes with statistical analysis of the densitometric analysis represented by the graphs (quantified by ImageQuant software). (I) Fluorescence examination of *SOAT1* activity by applying NBD-cholesterol as the substrate. EECs were incubated with 10 μg/mL of NBD-cholesterol at 37°C for 30 mins or 1 hour. Green indicated the cholesterol ester inside of EECs and blue indicated cell nucleus stained with DAPI. (J-K) The fluorescent intensity was quantified by ImageJ software. EECs were treated with or without db-cAMP for 24 hours. EECs were then incubated with 10 μg/mL of NBD-cholesterol for 30 min or 1 hour and examined by confocal microscopy. (J) Fluorescent intensity of total area. K: Fluorescent intensity per area of lipid droplets in EECs. (n = 4) Statistical significance was determined by one-way analysis of variance. Tukey‘s test was used to evaluate differences between means. Control value was set as 1. Different letters indicate a significant difference (P≤0.05). (L) The *SOAT1* protein levels after db-cAMP or IBMX treatments. EECs were treated with 0.5 or 1 mM db-cAMP or 0.5 mM IBMX for 48 hours and protein levels were examined by Western blotting. The *β-actin* was used as internal control. Statistical significance was determined by one-way analysis of variance. Dunnett's multiple comparisons test was used to evaluate differences between means. Data were expressed as means ± S.E.M. (n = 8) and different letters indicate the significant difference (P≤0.05). (M-N) The effects of H89 with or without db-cAMP and IBMX treatments on NBD-cholesterol uptake and storage. (M) EECs were pre-treated with or without H89 (10 μM) for 2 hours, and added db-cAMP (1 mM) or IBMX (0.5 mM) for 24 hours. Cells were then incubated with 10 μg/mL NBD-cholesterol for one hour, fixed with 4% paraformaldehyde and finally examined by confocal microscopy. Green indicated the cholesterol ester inside of EECs and blue indicated cell nucleus stained with DAPI. Scale bar = 50 μm. (N) The quantitation of fluorescent intensity on NBD-cholesterol uptake and storage by ImageJ software in cells treated with H89 with or without IBMX or db-cAMP. Data were expressed as means ± S.E.M. (n = 4) Statistical significance among more than three different experimental groups was determined by one-way analysis of variance. Tukey‘s test was used to evaluate differences between means. Different letters indicate a significant difference (P≤0.05).

In Fig 1, all the experiments represented 6 separated repeats, except for 1A, in which 6 to 9 repeats were performed. In Fig 2, four repeats were performed to generate the data, except for 2E and 2F, in which 6 repeats were performed.

### 2. Real-Time PCR for measuring gene mRNA accumulations

Total RNA was extracted from cultured cells using GENEzol™ Reagent (New Taipei City, Taiwan) according to the manufacturer’s protocol. Total RNA was reverse-transcribed with random primers using the high capacity cDNA re-verse-transcription kit (#4368814, ThermoFisher, Waltham, MA, USA). The mRNA accumulation was determined using the SYBR green reagent (ThermoFisher). Real-Time PCR was performed using the Step One Plus Real-Time PCR System (ThermoFisher) with the following incubation protocol: initial 7 minutes at 95°C, followed by 39 cycles of 10 seconds at 95°C, 30 seconds at approximately 60°C (see Table 1), with a final extension for 1 minute at 60°C. The *β-actin* mRNA in the same sample served as internal control. Threshold cycle (Ct) values were obtained and relative gene expression was calculated using the formula: 2^-(Ct target genes- Ct β-actin)^ [17]. The sequences of primers were indicated in Table 1.

**Table 1:** The chicken primers for real-time PCR quantification.

Gene	Primer sequence (5’→3’)	Product size (bp)	Annealing temp. (°C)
ch-SOAT1 (XM_015290507.1)	S 5’-GAAGGGGCCTATCTGGAACG-3’ A 5’-ATCTGCACGTGACATGACCA-3’	168	59.7
ch-CREB1* (NM_204450.2)	S 5’-CGGCGGAGGTGTAGTTTGAC-3’ A 5’-TTTCTGCCTCTGTAACGGCT-3’	176	57.6
ch-CREBBP** (XM_015294628.1)	S 5’ -AGCGGCTCTTCCTTAAACCC-3’ A 5’ -CCCTGGTTTAACGGGCTCTT-3’	146	58.8
ch-β-actin (NM_205518.1)	S 5’-GTGATGGACTCTGGTGATGG-3’ A 5’-TGGTGAAGCTGTAGCCTCTC-3’	151	60

*CREB1: cAMP responsive element binding protein 1

** CREBBP: phosphorylated CREB binding protein.

### 3. Analysis of *SOAT1* activity

To determine whether *SOAT1* activity can be regulated through a cAMP-dependent pathway, EECs were cultured with a cholesterol analog, 22-(N-(7-Nitrobenz-2-Oxa-1,3-Diazol-4-yl) Amino)-23,24-Bisnor-5-Cholen-3β-Ol (NBD cholesterol, N1148, ThermoFisher); when NBD-cholesterol is esterified by *SOAT1*, expression of green fluorescent protein is greatly increased. After EECs on glass slides were cultured with db-cAMP for 24 hours, 10 μg/mL NBD-cholesterol was added for an hour and then EECs were rinsed with 4% paraformaldehyde overnight and then washed twice with PBS. Cell nucleus were stained with DAPI counterstain (30–804930, Abbott, Des Plaines, IL) for 10 minutes and then washed with PBS. Stained EECs were mounted on glass slides with UltraCruz® aqueous mounting medium (sc-24941, Santa Cruz Biotechnology, Dallas, Texas,USA) and examined using the Leica TCS SP5 II confocal microscope (488 nm excitation, 540 nm emission). Then the *SOAT1* activity was measured [18].

To confirm the inhibitory effect of H89 on *SOAT1*’s function on NBD-cholesterol uptake and storage, EECs on glass slides were pre-treated with H89 (10 μM, 10010556, Cayman) for 2 hours before db-cAMP (1 mM) or IBMX (0.5 mM) activation for 24 hours. Then NBD-cholesterol was added for one hour, as above. After 24 hours, cells were washed with PBS and fixed with 4% paraformaldehyde overnight. The slides were examined for green fluorescence by confocal microscopy.

### 4. SDS-polyacrylamide gel electrophoresis and immunoblotting

EECs were treated with db-cAMP (0.5 and 1 mM) or IBMX (0.5 mM) for 48 hours, and total protein was extracted with 1X RIPA buffer (20–188, Merck, Darmstadt, Germany), supplemented with Pierce^TM^ proteinase and phosphatase inhibitor mini tablets (A32959, ThermoFisher). The results in Fig 2L represented 8 repeats for EEC culture experiment. After homogenization procedure and preliminary centrifugation (17K x g at 4°C for 15 minutes) to remove mitochondria, cell membranes, nucleus and intact cells, the supernatant from EECs were sedimented at 145K x g for 2 hours (CP80WX, Hitachi Koki Co., Ltd., Hitachi, Tokyo, Japan) to collect microsomes, then were sonicated and stored in -20°C for Western blotting following the previously described procedure [19]. In brief, microsomes (10 μg protein/sample as determined by the method of Bradford reagent (B6916, Sigma-Aldrich, according to the manufacturer’s protocol)) were subjected to 10% SDS-PAGE gel, and the separated proteins were electrophoretically transferred to a polyvinylidene difluoride (PVDF) membrane (NEF1002001PK, PerkinElmer, Waltham, MA, USA). Nonspecific binding sites were blocked with gelatin buffer (0.25% Gelatin, 0.5 M NaCl, 5 mM EDTA-2Na, 0.05% Tween-20, 50 mM Tris (pH8.8)) for 1 hour at room temperature. *SOAT1* was detected with rabbit anti-mouse *SOAT1* primary antibody (antibody diluted 1:500, orb100781, Biorbyt, Cambridgeshire, UK) followed by incubation with anti-rabbit IgG HRP-linked secondary antibody (1:5000, 7074P2, Cell Signaling, Danvers, MA, USA). The *β-actin* protein (antibody diluted 1:5000, sc-4778, Santa Cruz) was detected as an internal control. Two proteins were detected with the enhanced chemiluminescence protocol (WBKLS0500, Immobilon western, Millipore, USA). The sizes of proteins were estimated with a PageRuler™ Prestained Protein Ladder (10–180 kDa) (26616LCS, ThermoFisher). Protein quantifications were performed with Bio-Rad ChemiDoc Touch Imaging program.

### 5. Analysis of *SOAT1* promoter activity by reporter gene activity

The chicken genomic DNA was collected from blood extracted with DNeasy blood & tissue kit (69506, QIAGEN, Valencia, CA, USA), according to the manufacturer’s protocol. We amplified the partial chicken *SOAT1* promoter by Phusion High-Fidelity DNA Polymerase (F530L, ThermoFisher). PCR reactions were prepared with a total reaction of 10 μL, including 2 μL of 5X Phusion HF buffer, 0.2 μL of phusion DNA polymerase, 0.8 μL of 5 mM dNTPs, 0.5 μL of 10 μM primer stock (Sense: 5’-CGGGATCCCACGTCACCGCGCA-3’; Antisense: 5’-CCTACGTACCATTTCTGACGCT-3’), 1 μL of genomic DNA (100 ng/μL), and 5.5 μL of ddH_2_O. The PCR regime was started with denaturation at 98°C for 30 seconds followed by 35 cycles of 98°C for 10 seconds, 63.5°C for 30 seconds and 72°C for 12 seconds. Terminal extension was for 10 minutes at 72°C. The PCR product (410 bp) was then separated by gel electrophoresis at 100 V for 40 minutes, followed by purification of the target PCR products (28706, QIAGEN).

We then used T4 DNA ligase (EL0011, ThermoFisher) and the CloneJET PCR Cloning kit-pJET1.2/blunt (K1232, ThermoFisher) to anneal the PCR product. The reactions were prepared in a total volume of 20 μL, containing 8 μL of PCR product (insert), 1 μL of pJET1.2/blunt (vector), 10 μL of 2X Reaction buffer, and 1 μL of T4 DNA ligase. Incubation was at room temperature for 15 minutes. The reactions were transformed into competent cells, DH5α (RH617, RBC Biosciences, Korea). We sequenced the successful transformed colonies. The pJET1.2/blunt-*SOAT1*p and pGL3-basic vector (pGL3-luciferase reporter vector, E1751, Promega, USA) were cut by restriction enzymes, XhoI (R0146S, NEB, UK) and BglII (R0144S, NEB, UK) at 37°C for 1 hour. Then the reactions were repeated with ligation, transformation and sequencing to obtain the pGL3-*SOAT1*p.

We used pGL3-*SOAT1*p as the positive control and pGL3-basic vector (pGL3-luciferase reporter vector, E1751, Promega, USA) as the negative control in the 293T cell experiment. 293T cells (CRL-11268, ATCC, Manassas, VA, USA) were seeded on 96-well plates (1.0×10^4^ cells/well) and cultured in DMEM containing with 10% FBS and 1% PSA. PolyJet^TM^ (SL100688, SignaGen, Rockville, MD) was used for transfection and quick screening for promoter activity. Twenty-five ng Renilla was used as internal control. The effects of 0.5 mM IBMX, 10 μM H89 and IBMX plus H89 on 293T cells were determined using the Dual-Glo Luciferase Assay system (E2940, Promega, Madison, WI) with a luminometer (Hidex, Turku, Finland) to quantify the *SOAT1* promoter activity. The peak emission wavelength is at 560 nm.

### 6. Serial deletions of selected transcription factor binding site on *SOAT1* promoter region

We amplified the plasmid of pJET1.2/blunt-*SOAT1*p (constructed in our lab) with primers that spanned the restriction enzyme cutting sites (KpnI & HindIII). PCR reactions were prepared with a total reaction of 10 μL, including 2 μL of 5X Phusion HF buffer, 0.2 μL of Phusion DNA polymerase (2 U/μL), 0.8 μL of 2.5 mM dNTPs, 0.5 μL of 10 μM primer stock (see Table 2.), 1 μL of genomic DNA (100 ng/μL), and 5.5 μL of ddH_2_O. The PCR regime was 39 cycles of 98°C for 10 seconds, various annealing temperatures (see Table 2.) for 30 seconds and 72°C for 12 seconds with final extension for 10 minutes at 72°C. The restriction enzymes KpnI (R0142S, NEB, UK) and HindIII (R0104S, NEB, UK) were then added to the PCR products and pGL3 plasmids (did not undergo PCR amplification) with incubation for 1 hour at 37°C. Digested products were then separated by gel electrophoresis at 100 V for 40 minutes, followed by purification of the target PCR products (28706, QIAGEN).

**Table 2:** Conditions and primers for serial promoter region deletion.

Items	Primer sequence (5’→3’)	Product size (bp)	Annealing temp. (°C)	Restriction enzyme
ch-SOAT1p-pGL3	S 5’-GCGCGGTACCCCATTTCTGACGCT-3’ A 5’-GCGCAAGCTTCACGTCACCG-3’	410	63.5	*Kpn*I *Hind*III
ch-SOAT1p-delete1	S 5’ -GCGCGGTACCTCGTTGAGC-3’ A 5’-GCGCAAGCTTCACGTCACCG-3’	300	57.0	
ch-SOAT1p-delete2	S 5’-GCGCGGTACCTTCCCTGCCAA-3’ A 5’-GCGCAAGCTTCACGTCACCG-3’	280	65.0	
ch-SOAT1p-delete3	S 5’-GCGCGGTACCAATGGGCG-3’ A 5’-GCGCAAGCTTCACGTCACCG-3’	71	59.4	

We then ligated the purified-PCR products with the luciferase-containing pGL3: 8 μL of PCR product (insert), 1 μL of pJET1.2/blunt (vector), 10 μL of 2X Reaction buffer, and 1 μL of T4 DNA ligase. The reactions were mixed well and incubated at room temperature for 15 minutes. Finally, the insert–vector ligate was transformed into competent cells, DH5α (RH617, RBC Biosciences, Korea). We sequenced the successful transformed colonies.

The 293T cells were seeded on 96-well plates (1.0×10^4^ cells/well). We examined the luciferase activities of serial promoter deletion plasmids (250 ng/each plasmid) and used the pGL3-basic vector as the negative control. PolyJet^TM^ (SL100688, SignaGen, Rockville, MD) was used for transfection and quick screening for promoter activity. Twenty-five ng Renilla was used as internal control. Cells were then cultured without serum for 6 hours and treated with 1 mM IBMX for activation. The luciferase activities were determined after 24 hours using the Dual-Glo Luciferase Assay system (E2940, Promega, Madison, WI) to quantify the *SOAT1* promoter activity.

### 7. Activity assay of *SOAT1* promoter mutation on predicted transcription factor binding sites

The PCR primer set was indicated in Table 3. The primers for ch-*SOAT1*p-pGL3 Mutant 1 were used for the first PCR (PCR-MU-1), and primers of ch-*SOAT1*p-pGL3 Mutant 2 were used for the second PCR (PCR-MU-2). The PCR-MU-1 and 2 reactions were: 10 μL of 5X Phusion GC buffer, 0.5 μL of Phusion DNA polymerase (1.0 units/ 50 μL reaction), 4 µL of 2.5 mM dNTPs, 1.5 μL of 100% DMSO, 2.5 μL of primer stocks (0.5 μM), 250 ng of template DNA, and nuclease-free water up to 50 μL. The products of these PCR’s were gel extracted and sequenced.

**Table 3:** Promoter mutation primer sets.

ch-SOAT1p-pGL3 Mutant 1	Sense 5’-GCGCGGTACACCGTTTACATCAATCCC-3’ Anti-sense 5’-GCGCAAGCTTCACGTCACCG-3’
ch-SOAT1p-pGL3 Mutant 2	Sense 5’-GCGCGGTACCCCATTTCTGACGCT-3’ Anti-sense 5’-GCGCAAGCTTGGGATTGATGTAAACGGTCACGTCACCG-3’

The template DNA for the final PCR was the pooled product of PCR-MU-1 and 2 (ratio 1:1). The ch-*SOAT1*p-pGL3 Mutant 1 A (anti-sense) primer was used as reverse primer, the ch-*SOAT1*p-pGL3 Mutant 2 R (sense) primer was used as forward primer.

The mutation PCR protocol was conducted as follows: initial denaturation at 98°C for 30 seconds, then 34 cycles of 98°C for 1 minute, 63.5°C for 30 seconds and 72°C for 1 minute with a final extension at 72°C for 10 minutes and cooling at 4°C. We transfected the mutations into 293T cells, and treated the cells with 0.5 mM IBMX and/or 10 μM H89 for 24 hours, then assayed the luciferase activity.

### 8. The mRNA accumulations of the predicted transcription factors

Based on the previous results, the location of the crucial transcription factor binding region was at promoter sequence -349 bp to -341 bp; and according to results of Genomatix prediction, the binding site would bind with *CREB1* (cAMP responsive element binding protein 1) and *CREBBP* (phosphorylated CREB binding protein). The EECs and hepatocytes were treated with IBMX and/or H89 for 24 hours, total RNA was extracted and the transcription factor levels were measured by real-time PCR; the primer sets were described in Table 1.

### 9. Statistical analysis

All data were analyzed by one-way analysis of variation. The major effect between treatments was determined by Tukey’s post-hoc test. The significance level used was at P ≤ 0.05.

## Results

### 1. Effects of nutrients on SOAT1 mRNA accumulation

We confirmed that *SOAT1* was in YSM by real-time PCR, and that the SOAT1 mRNA was expressed in YSM during entire embryonic development (Fig 1A). Therefore, we suggested that *SOAT1* may have significant roles during development. Palmitic acid (PA) and oleic acids (OA) are the two most abundant fatty acids in egg yolk both in Japanese quail and chicken egg yolk. In quail eggs, there is approximately 17% of PA (C16:0) and 27% of OA (C18:1n9c); in chicken eggs, there is approximately 27% of PA and 30% of OA [20, 21]. To determine the effects of fatty acids on *SOAT1* transcriptions, we treated cells with two long-chain fatty acids which had the greatest concentration in avian yolk. Addition of OA or PA had no effects on the mRNA accumulation of *SOAT1* in either EECs (Fig 1B) or primary hepatocytes (Fig 1C). Higher glucose concentrations, including 3.3 mM, 5.5 mM and 17.5 mM, all inhibited the mRNA accumulation of *SOAT1* in hepatocytes (Fig 1E). Treatment with 3.3 mM glucose, but not higher concentrations elevated *SOAT1* mRNA accumulation in EECs (Fig 1D).

### 2. Effects of IBMX and hormones on the mRNA accumulation of *SOAT1* in EECs and hepatocytes

The IBMX blocks phosphodiesterase and enhances intracellular cAMP concentrations [12]. Treatment with 0.5 mM IBMX or 100 nM glucagon increased the mRNA accumulation of *SOAT1* in both EECs (Fig 1F and 1H) and hepatocytes (Fig 1G and 1I). To demonstrate whether the effects were acting through a cAMP-dependent pathway, we challenged EECs and hepatocytes with forskolin or dibutyl-cAMP (db-cAMP). Fifty μM forskolin or 1 mM dibutyryl-cyclic AMP promoted the mRNA accumulation of *SOAT1* in both EECs (Fig 2A and 2C) and hepatocytes (Fig 2B and 2D), suggesting that regulation of *SOAT1* gene expression was through cAMP-PKA signaling. We added H89, a PKA inhibitor to show that these effects were neutralized (Fig 2E and 2F). Using the PepTag® Assay, we found that PKA phosphorylation was induced by IBMX, and this effect was neutralized by H89 (Fig 2G and 2H), therefore, both cAMP and IBMX up-regulated the expression of *SOAT1* by activating PKA.

### 3. *SOAT1* activity is regulated by cAMP

We used NBD-cholesterol to identify the esterification of cholesterol and to estimate the enzymatic activity of *SOAT1* in EECs (Fig 2I). The expression of the fluorescent signal was quantified by ImageJ Software (Fig 2J). Supplementation with db-cAMP in the culture medium increased the activity of *SOAT1* to synthesize cholesteryl esters (Fig 2I) and enhanced the cholesteryl ester accumulation in cells (Fig 2K). We confirmed that the protein levels of *SOAT1* were significantly increased by db-cAMP treatment (Fig 2L), implying that treatments of cAMP activated not only mRNA accumulation, but also *SOAT1* protein in EECs.

Treatment with H89 reduced *SOAT1* transcript accumulation. To analyze the H89 effect on *SOAT1*’s function, we treated with H89 before cAMP activation in EECs, and found that H89 had significantly reduced uptake of NBD-cholesterol (Fig 2M and 2N). Treatment with db-cAMP rescued the inhibition caused by H89 in EECs.

### 4. The cAMP-dependent response elements on the avian *SOAT1* promoter region

**Fig 3:**
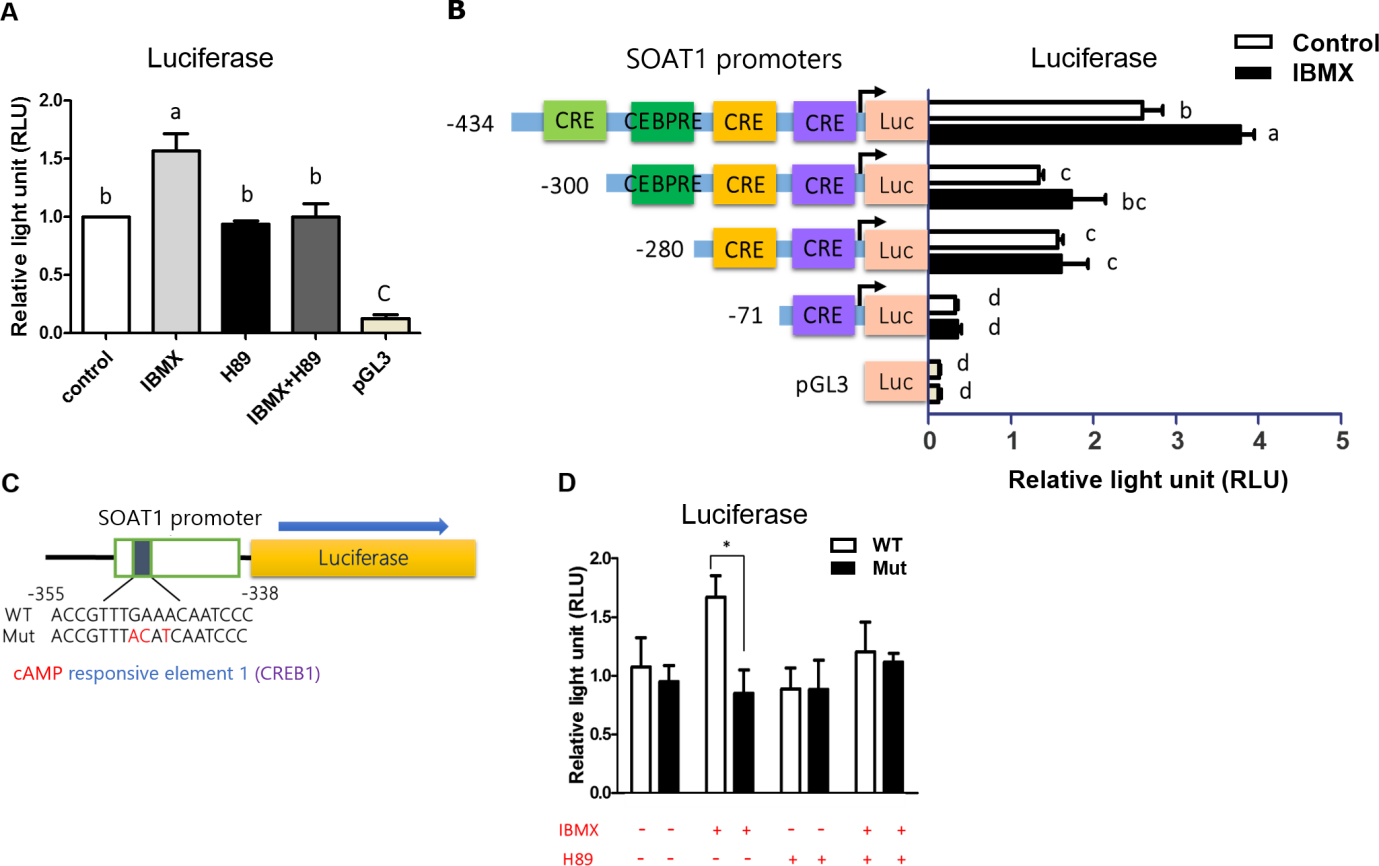
The importance of functional responsive elements CRE (cAMP-responsive element) on the *SOAT1* promoter. (A) Effect of IBMX and the PKA inhibitor, H89 on the induction of *SOAT1* promoter activity. Promoter activities were normalized for transfection efficiency using the renilla luciferase activity. (n = 5) (B) Effect of IBMX on the induction of serially deleted promoter regions of *SOAT1*. The 293T cells were transfected with variable length constructs of the *SOAT1* promoter and promoter activities were assayed after 24 hours of IBMX treatment. The pGL3 was a luciferase vector without *SOAT1* promoter insertion, and was used as a negative control. (n = 6) (C) Diagram of wild type and mutated cAMP-responsive element on the *SOAT1* promoter construct in a luciferase vector. (D) Effect of IBMX and H89 on the induction of promoter activity by the mutated CREB in the *SOAT1* promoter. The 293T cells were transfected with normal or mutated *SOAT1* promoter, and promoter activities were assayed after 0.5 mM IBMX and 10 μM H89 (PKA inhibitor) treatments for 24 hours. (n = 6) Data were expressed as means ± S.E.M. (n = three independent experiments) Statistical significance was determined by one-way analysis of variance. Tukey's test was used to evaluate differences between means. Control value was set as 1. Different letters indicate a significant difference (P≤0.05).

The serial promoter deletion study was designed to ascertain the location of regulatory function for gene transcription. IBMX treatment significantly increased the reporter signal of the full length promoter activity, and H89 treatment inhibited the IBMX effect (Fig 3A), suggesting that the regulatory activity of IBMX was working through a cAMP-dependent pathway. A deletion of the promoter region from -434 to -300 bp reduced the overall promoter activity, whereas the ability of IBMX to regulate function was still somewhat effective (Fig 3B). Deletion of the region from -287 to -71 bp eliminated all promoter activity which implied that the basal promoter area was located in this region (Fig 3B). To confirm the significant role of the sequence between -434 bp to -300 bp (inside of a CRE, cAMP responsive element), we mutated (Fig 3C) the region from -349 to -341 bp (accgtt**TGAAacaat**ccc). The mutated sequence had lower activity to drive expression of *SOAT1* when induced by IBMX (Fig 3D), suggesting that this specific sequence is important in mediating the transcriptional regulatory function of *SOAT1*.

### 5. The accumulation of *CREB1* and *CREBBP* is regulated by IBMX

**Fig 4:**
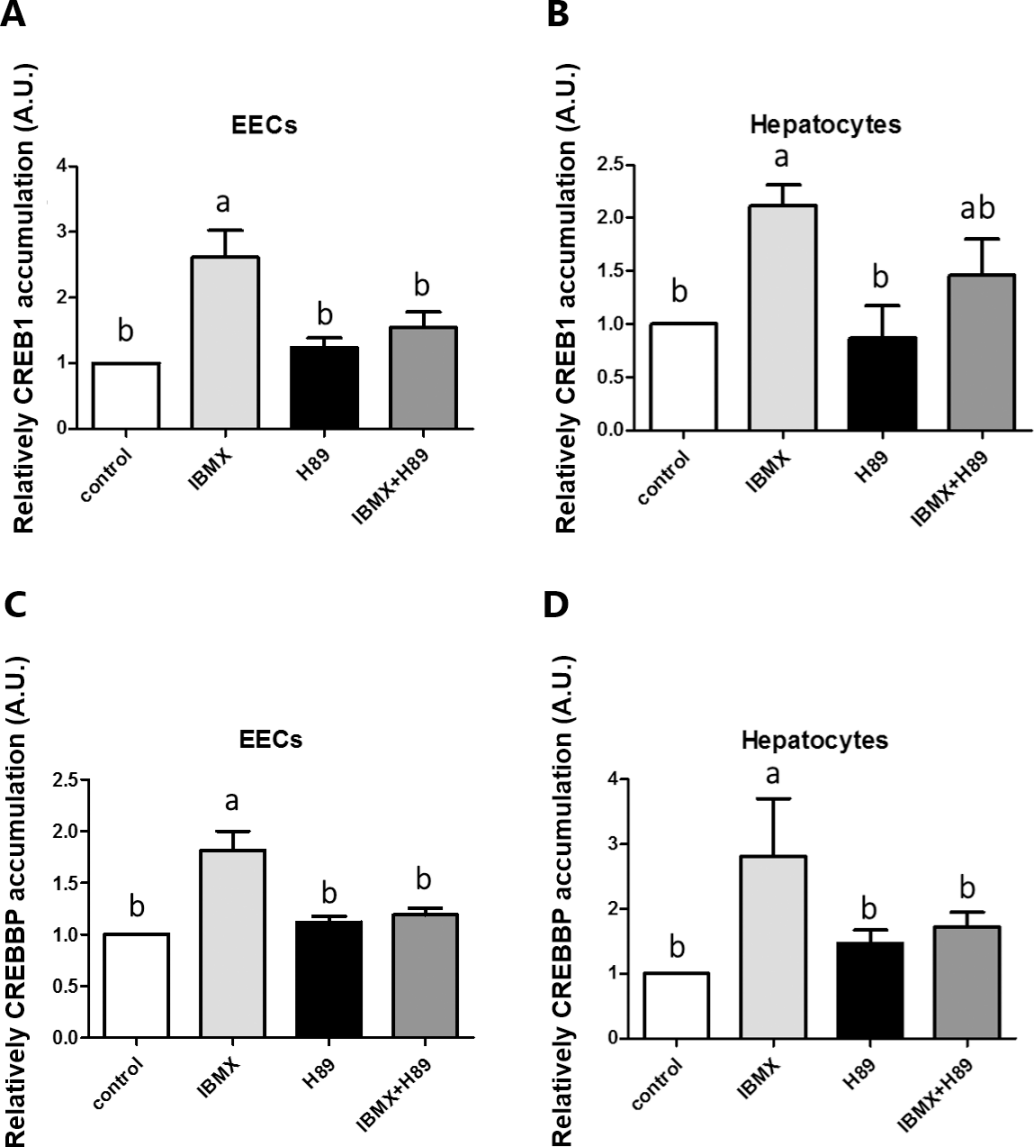
H89 inhibited IBMX-induced transcription factors mRNA accumulations in EECs and hepatocytes. Cells were treated with 0.5 mM IBMX alone for 24 h, *CREB1* and *CREBBP* mRNA accumulations were increased, whereas the H89 (PKA inhibitor, 10 μM) abolished the increased mRNA accumulations mediated by IBMX in both culture systems. Data were expressed as means ± S.E.M. (n = 6) Statistical significance was determined by one-way analysis of variance. Tukey's test was used to evaluate differences between means. Control value was set as 1. Different letters indicate a significant difference (P≤0.05).

Because the promoter sequence of -349 to -341 bp can interact with *CREB1*, we measured the accumulation of *CREB1* and *CREBBP* mRNA with or without IBMX treatment in quail EECs (Fig 4A and 4C) and hepatocytes (Fig 4B and 4D). The IBMX treatment increased accumulation of both *CREB1* and *CREBBP* mRNA compared with the control group. Adding H89 to inhibit PKA activity reversed the IBMX effect, suggesting that *CREB1* and *CREBBP* was also regulated by IBMX through the PKA pathway.

## Discussion

The fats in yolk provide energy and lipid components for avian embryos, especially for the final stages of fast dynamic growth during the last week of development. Between embryonic day (ED) 13 to ED17, the amount of fat in YSM increased in chicken embryos and for four days before hatching (from ED17 to ED21), there was a significant decrease of YSM fat [22]. This may reflect the occurrences of YSM proliferation between ED13 to ED17 and degeneration from ED17 to ED21. In addition, nutrient transfer from yolk to the circulatory system is critically dependent on the YSM, and the consumption of the yolk sac is enhanced before hatch (direct transport from yolk stalk to embryo’s intestine), especially from ED19 to ED21 [22]. The high capacity of the area vasculosa of YSM to perform lipid hydrolysis and re-esterification of free fatty acids to form acyl-lipids during the transfer process in YSM is known [23].

The main players in cholesterol esterification are the *SOAT* family. In the gut, the *SOAT* family is partially responsible for cholesterol absorption [24]; in the adrenal cortex, the rate of cholesterol esterification regulates cholesterol availability for steroid production [25]. Furthermore, hepatic cholesterol esterification regulates, in part, biliary cholesterol secretions and VLDL cholesteryl ester secretions [10]. In the current study, we found that increased cAMP up-regulated the promoter activity and transcription and protein levels of *SOAT1* in YSM during avian embryonic development. Glucagon and IBMX are factors that can increase the cAMP concentration. Although the basic plasma glucagon concentration in late embryogenesis is no more than 0.12 nM (400 pg/mL) [26], much lower than the dose we used in the EECs and hepatocyte experiments, treatment of avian embryos with exogenous glucagon may be a tool to modify *SOAT1* activity to increase lipid utilization.

The level of *SOAT1* is involved in cholesterol homeostasis and regulatory function of sterol regulatory element-binding protein 1 (*SREBP-1*) in lipogenesis [27]. Therefore, we suggest that *SOAT1* regulation can be important to sustain normal lipid utilization in avian embryonic development. Understanding the regulatory mechanisms of *SOAT1* is a critical step to develop a strategy to modify YSM *SOAT1* transcription and function to improve embryonic development and survival rate.

High mRNA accumulation and activity of *SOAT1* in avian YSM [2, 4] and the large amount of newly formed cholesteryl esters in YSM [2, 4, 28] indicate that cholesterol absorption from yolk to EECs, is followed by esterification to cholesteryl esters for further transport into embryos. The *SOAT1* activity is up-regulated by liver cytosolic proteins and low molecular weight proteins (12–15 kDa) from chicken livers under the influence of fatty acid binding protein (FABP) [29]. Intake of different dietary fats with or without cholesterol results in modified microsomal *SOAT1* activity in livers of rabbits [8]. In these rabbits, hepatic *SOAT* activity is increased by fish oil diets (enriched in n-3 fatty acids), owing to the change in membrane fatty acid composition that may alter the availability of cholesterol to the enzyme [8]. A 2% cholesterol-enriched diet results in a significant increase in *SOAT* activity and the rate of cholesteryl ester formation; therefore, cholesterol substrate availability is a major regulator of *SOAT* activity [10]. In spite of the speculations above, we found no differential *SOAT1* mRNA accumulation after cholesterol treatment (29.5 or 129 μM) in in vitro experiments (data not shown).

For yolk sac retraction during the onset of lung respiration and hatching, the thyrotropic, corticotropic and somatotropic axes undergo massive changes [30]. Plasma growth hormone, somatostatin and thyrotropin releasing hormone are elevated before hatching [31]. Plasma T4 levels increase gradually throughout the last week of embryonic development and reach a maximum around hatching. Plasma T3 levels remain very low during most of embryonic life, whereas they markedly increase around the transition from chorioallantoic to pulmonary respiration [26, 30]. There is a significant increase in plasma insulin with increasing age from ED 10 to hatch. Plasma glucagon levels remain low until ED 17, and then significantly increase approximately 3-fold at hatch, which corresponds with increasing plasma glucose levels during late embryo development [26]. We suspected that avian *SOAT1* activity would also be affected by these various hormones. We found no effect of estrogen or the glucocorticoid, dexamethasone, but found that glucagon stimulated SOAT1 mRNA accumulation and that exogenous glucose inhibited this activity in hepatocytes. We did find stimulation of *SOAT1* mRNA accumulation, but only at 3.3 mM added glucose in EEC.

The regulatory mechanism for the expression of avian *SOAT1* is still only partially elicited. We established serial promoter deletion studies based on regulatory sequence prediction and used cell culture models to study the molecular mechanism by which nutrients and hormones regulate the mRNA accumulation of *SOAT1*.

In a previous study, we utilized an embryonic endoderm epithelial cell culture system to demonstrate that *SOAT1* may be regulated by the cAMP dependent protein kinase A related pathway [14]. Cholesteryl ester accumulation was affected by reversible phosphorylation (the CREB site) with cAMP involvement, and in contrast, glucagon or dibutyrl cyclic AMP inhibited neutral cholesteryl ester hydrolase activity in rat primary hepatocytes [32]. Because glucagon and IBMX enhance the mRNA accumulation of *SOAT1*, we hypothesized that *SOAT1* can be regulated through cAMP-dependent protein kinase A. Low levels of glucose increased the mRNA accumulation of *SOAT1*, consistent with glucagon treatment, suggesting that the function of *SOAT1* may be regulated through a cAMP-PKA pathway. Moreover, there are several CRE response elements in the promoter sequence of avian *SOAT1*. Active PKA facilitates *CREB1* binding to a cAMP-responsive element [33]. Thus, we speculated that glucagon regulates the accumulation of *SOAT1* by increasing the cAMP level. In our finding, the IBMX (0.5 mM) treatment presumably increased cAMP to activate PKA which then activated SOAT1 mRNA accumulation in EECs. Numerous molecules have been demonstrated to be able to increase the cytosolic level of cAMP. For example, theophylline and caffeine inhibit phosphodiesterase to reduce the break-down of cAMP and increase intracellular cAMP concentration [34, 35]. Based on our present finding, the increase of cAMP (presumed because of the effect of IBMX and glucagon to activate PKA) can promote the activity of PKA to enhance the transcription and increased the function of *SOAT1*.

In THP-1 macrophages, the expression of *SOAT1* is regulated by insulin through mitogen-activated protein kinase (MAPK) [36]. The insulin treatment increases MAPK phosphorylation, and further increases the binding of CCAAT/enhancer-binding protein α (*C/EBPα*) to the promoter of *SOAT1*, suggesting that insulin can exert its function by regulating MAPK to modify *SOAT1* mRNA accumulation. Therefore, clarification of the regulatory mechanism for the expression of *SOAT1* in EECs during embryonic development will provide new strategies to increase hatchability of avian species.

**Fig 5:**
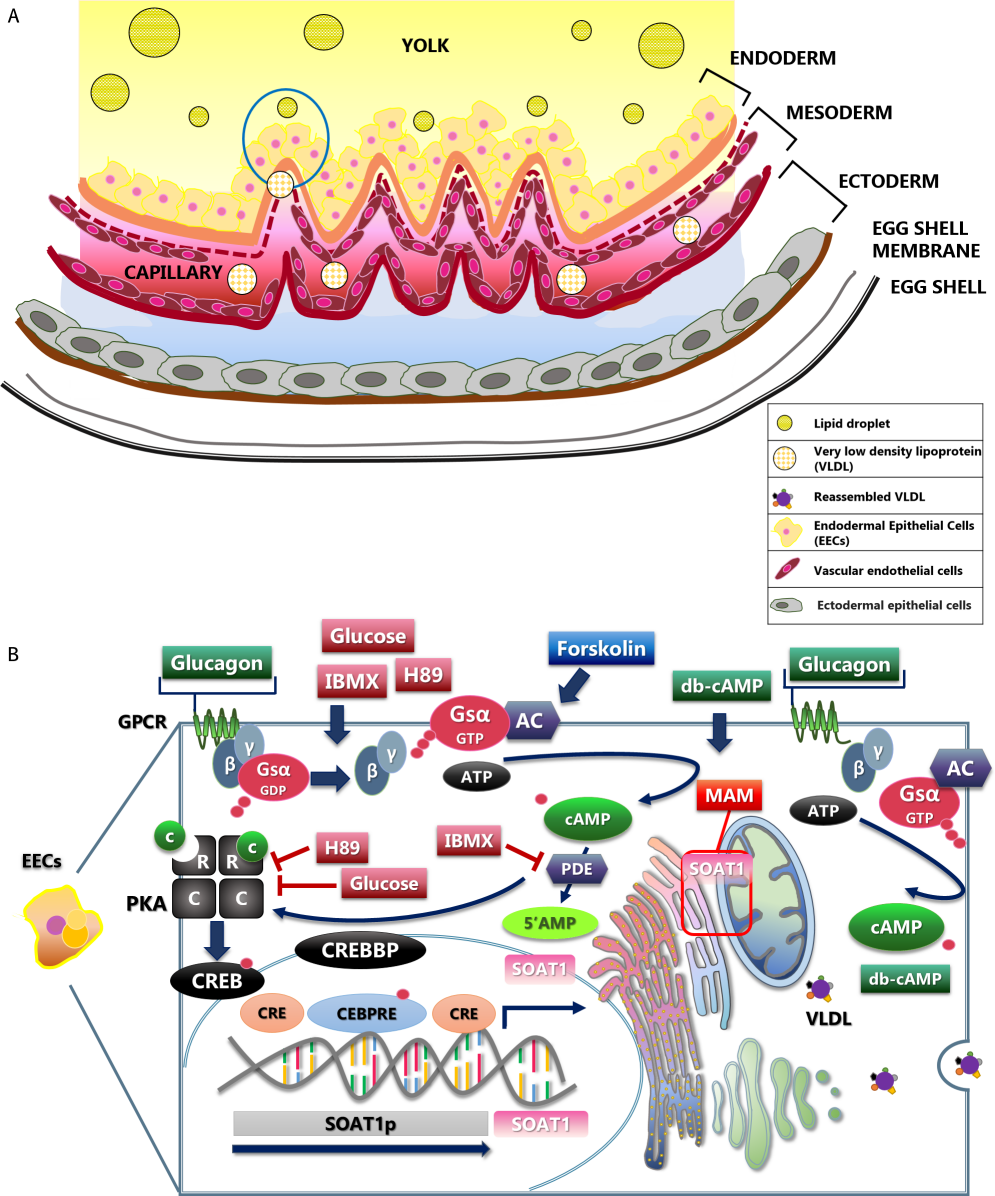
The illustration of YSM structure and the positive regulation of *SOAT1* enhancement by a cAMP-dependent pathway in avian EECs. AC, adenylyl cyclase; CRE, cAMP-response element; CREB1, cAMP responsive element binding protein; CREBBP, phosphorylated CREB binding protein; CEBPRE, C/EBP (CCAAT-enhancer-binding protein) response element; db-cAMP, dibutyryl-cAMP (a cAMP analog); MAM, mitochondria-associated membrane; PKA, protein kinase A; R-regulatory units, C-catalytic units. (Fig 5 was drawn by Han-Jen Lin).

In sum, the current study found that IBMX and glucagon increase the mRNA accumulation of *SOAT1* through a cAMP-dependent pathway, and promote the function of *SOAT1* to enhance cholesteryl ester formation. Therefore, we have established a possible regulatory mechanism by which these agents regulate the mRNA accumulation of *SOAT1* (**Fig 5A and 5B**). Whether an increase in function of *SOAT1* would enhance the hatchability of avian species is unknown. However, increasing *SOAT1* activity will certainly help to enhance lipid utilization and transportation during embryonic development.

## Conclusion

We found that *SOAT1* was regulated by glucose, glucagon and IBMX in avian EECs and hepatocytes. The mechanism by which these agents regulate the mRNA expression of *SOAT1*, is through the cAMP-dependent PKA pathway. Thus, an increase in the cAMP-dependent PKA activity is a possible strategy to improve the utilization of lipids in the EECs and to improve avian embryonic growth by regulation of the function of *SOAT1*.
